# Intraductal Tubulopapillary Neoplasm of the Pancreas Presenting as a Localized Pancreatic Tumor in a 52-Year-Old Woman: Focusing on a Rare Pancreatic Malignancy and Contrasting to Intrapapillary Mucinous Neoplasm

**DOI:** 10.7759/cureus.8548

**Published:** 2020-06-10

**Authors:** Edward Nabrinsky, Charisse Liz Baste, Miguel Gonzalez, Edward James

**Affiliations:** 1 Internal Medicine, Advocate Lutheran General Hospital, Park Ridge, USA; 2 Pathology, University of Illinois at Chicago, Chicago, USA; 3 Pathology, Advocate Lutheran General Hospital, Park Ridge, USA; 4 Medical Oncology, Advocate Lutheran General Hospital, Park Ridge, USA

**Keywords:** pancreas, pancreatic cancer, intaductal, malignancy, mucin, mucinous

## Abstract

Intraductal tubulopapillary neoplasm (ITPN) is a distinctive type of pancreatic tumor first discovered more than three decades ago. ITPNs currently account for less than 1% of all pancreatic exocrine tumor cases recognized, and less than 5% of pancreatic intraductal tumors. A patient’s presentation is often nonspecific in comparison to other intraductal pancreatic neoplasms. We discuss a 52-year-old female presenting with abdominal pain and weight loss with ITPN, and go on to define the typical presentation, clinical features, and pathology behind the tumor.

## Introduction

Intraductal tubulopapillary neoplasm (ITPN) is becoming more frequently recognized as a newer type of pancreatic tumor since the discovery of intraductal papillary mucinous neoplasm (IPMN) more than three decades ago. First recognized by Japanese researchers in the 1990s, ITPN is becoming its own entity with distinct clinical and pathological features. The 4th edition of the WHO defined ITPN as an intraductal tubule-forming epithelial neoplasm that possessed high-grade dysplasia and ductal differentiation but without overt production of mucin [1]. ITPN currently account for less than 1% of pancreatic exocrine tumor cases recognized, and less than 5% of all pancreatic intraductal cases [1]. We report a 52-year-old female presenting with abdominal pain and weight loss, with imaging demonstrating a mass primarily in the head of the pancreas as well as the uncinate process. After undergoing neoadjuvant chemotherapy, the patient underwent pancreatectomy with tumor resection and was discovered to have a 14.9 x 5.6 x 1.9 cm pancreatic mass. Histology confirmed the diagnosis of an ITPN. Herein, we explore and discuss the typical presentation, clinical features, and pathologic features of this tumor, as well as contrast this tumor to IPMN.

## Case presentation

**Figure 1:**
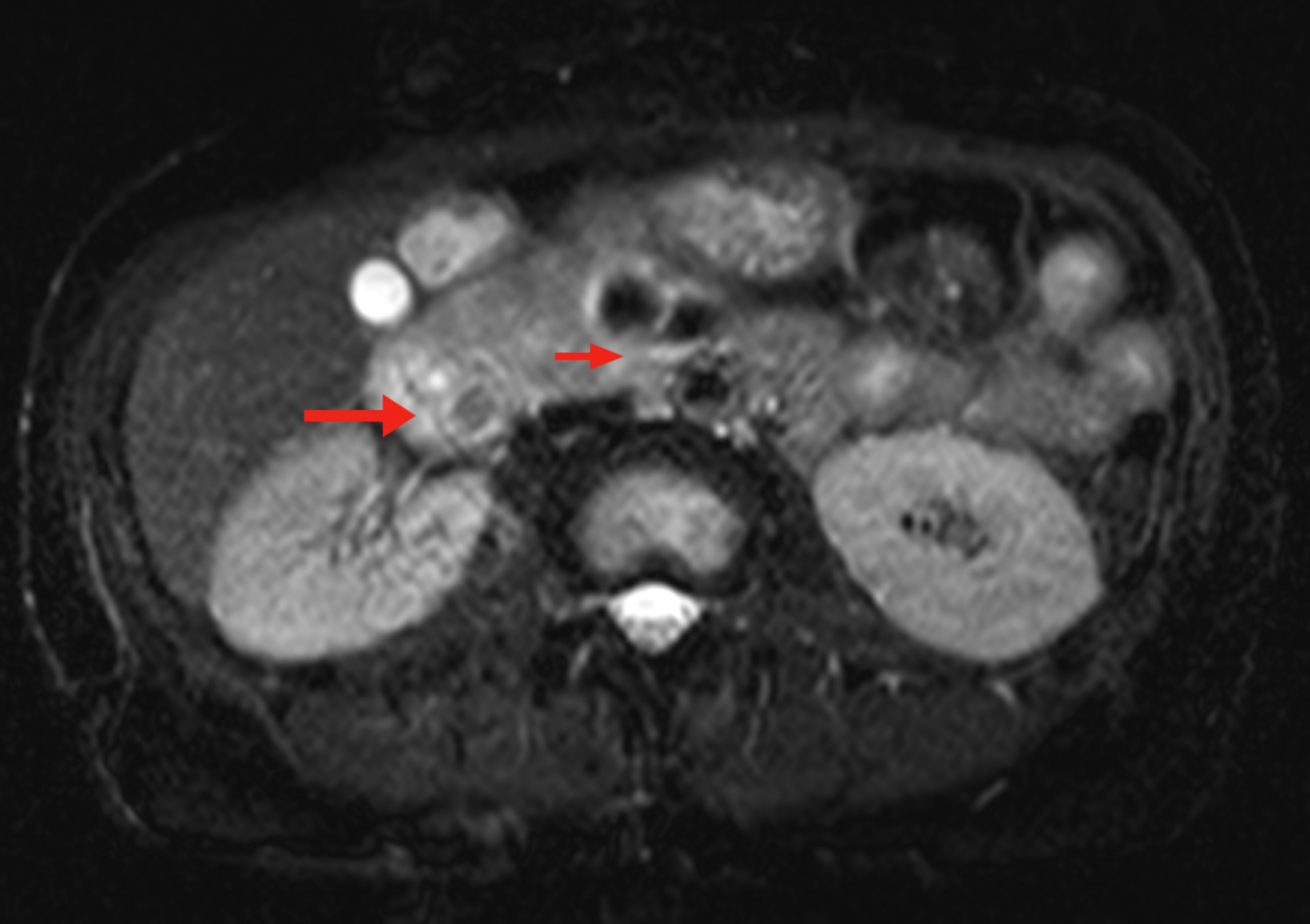
MRI of the pancreas on presentation Shown are the pancreatic head mass (large arrow) along with dilatation of the pancreatic duct (small arrow).

A 52-year-old female with a past medical history of type two diabetes mellitus and rheumatoid arthritis presented to the ER with intermittent abdominal pain, nausea, vomiting, and generalized weakness. She also endorsed losing 50 pounds unintentionally over the previous two months. The patient denied alcohol use but smoked one pack of cigarettes every two weeks. Imaging on presentation with CT scan of the abdomen and pelvis without contrast demonstrated a mass in the head of the pancreas; follow-up MRI of the abdomen demonstrated an irregular, enhancing mass in the head of the pancreas and uncinate process, measuring 3.3 x 3.2 x 3.6 cm, with corresponding dilatation of the pancreatic duct measuring up to 1.0 cm (Figure 1). Labs showed elevations of carbohydrate antigen 19-9 of 49 units/mL (reference range 0-35), carcinoembryonic antigen of 4.7 ng/dL (0-5.0), and alpha-fetoprotein (AFP) of 12 ng/dL (0-9). Hepatitis C antibody was positive and hepatitis C viral load was undetectable.

**Figure 2:**
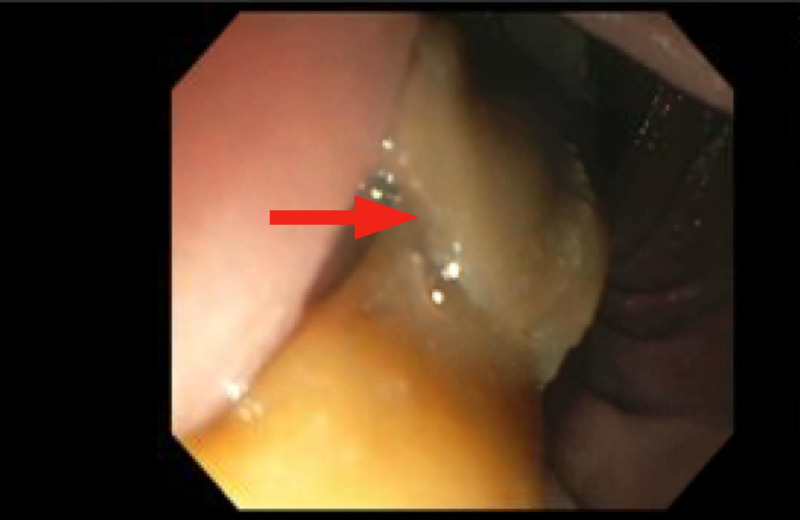
Endoscopic view of the pancreatic head mass (arrow)

The patient had a subsequent endoscopic ultrasound that confirmed a 2.5 x 3.0 cm mass in the pancreatic head and uncinate process (Figure 2), with maximal pancreatic duct diameter of 0.7 cm and abutment of the superior mesenteric vein. The mass was hypoechoic and heterogeneous with poorly defined endosonographic borders. Staging evaluation revealed localized disease and no evidence of metastasis. She underwent four cycles of neoadjuvant chemotherapy with gemcitabine and nab-paclitaxel and tolerated chemotherapy well with no significant side effects. Follow-up imaging showed a reduction in size of the primary tumor to 1.7 x 2.4 cm. She underwent pancreatectomy, splenectomy, subtotal gastrectomy, and feeding jejunostomy tube placement.

**Figure 3:**
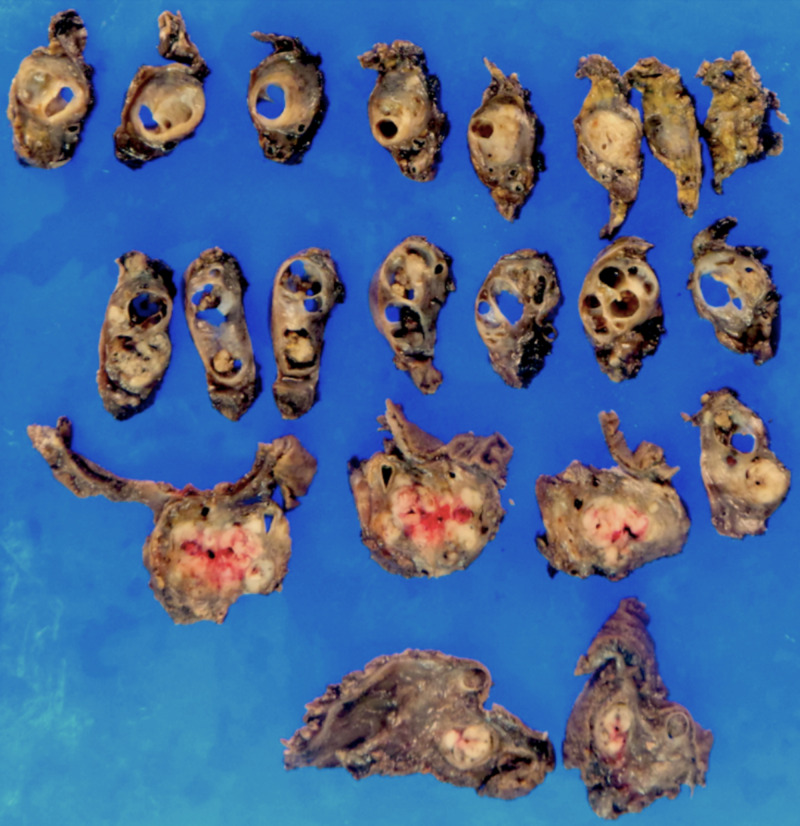
Gross pathological specimen Various segments of the resected mass demonstrating both heterogeneous focally necrotic as well as solid and cystic components

On pathological review, the tumor represented a 13.5 x 3.9 x 3.2 cm white-tan to yellow mass (Figure 3). This was larger than anticipated, based on the most recent imaging studies after completion of neoadjuvant chemotherapy. The mass involved the uncinate process as well as the body, neck and tail of the pancreas but was grossly contained within the gland.

**Figure 4:**
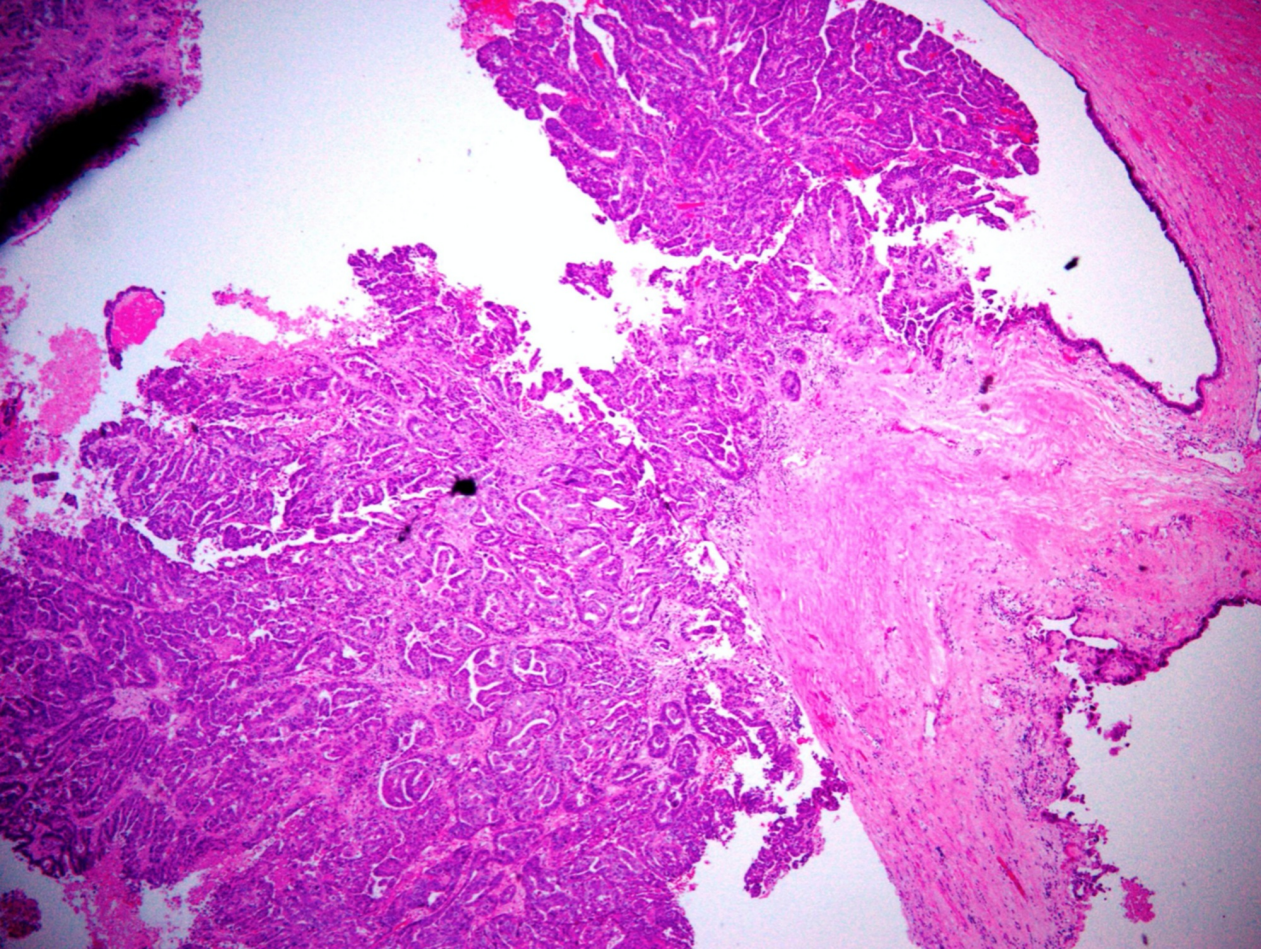
Papillary structures around fibrovascular cores

**Figure 5:**
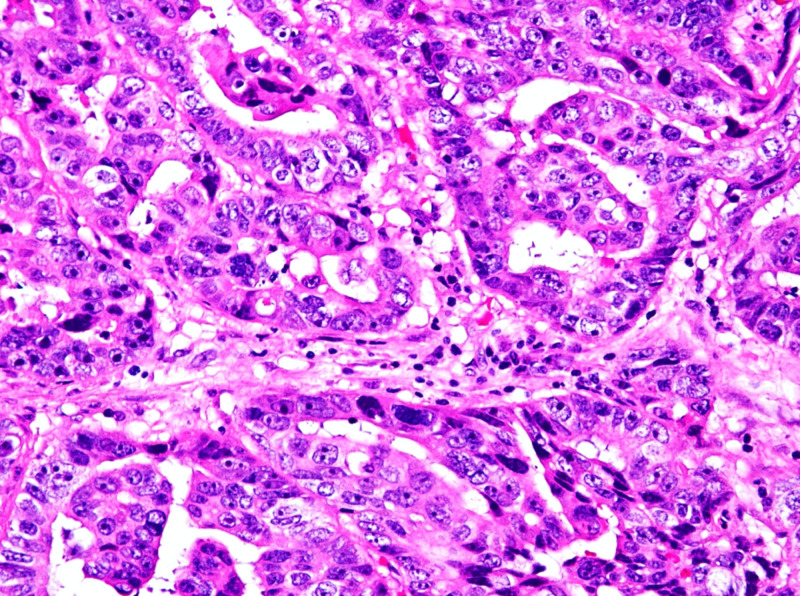
Highlighting eosinophilic cytoplasm

Histologically, the cystic areas displayed a cuboidal epithelial lining with polypoid proliferation of cuboidal cells forming ducts and complex papillary structures growing around fibrovascular cores (Figure 4). The cells exhibited pleomorphic nuclei, prominent nucleoli, and eosinophilic cytoplasm (Figure 5).

**Figure 6:**
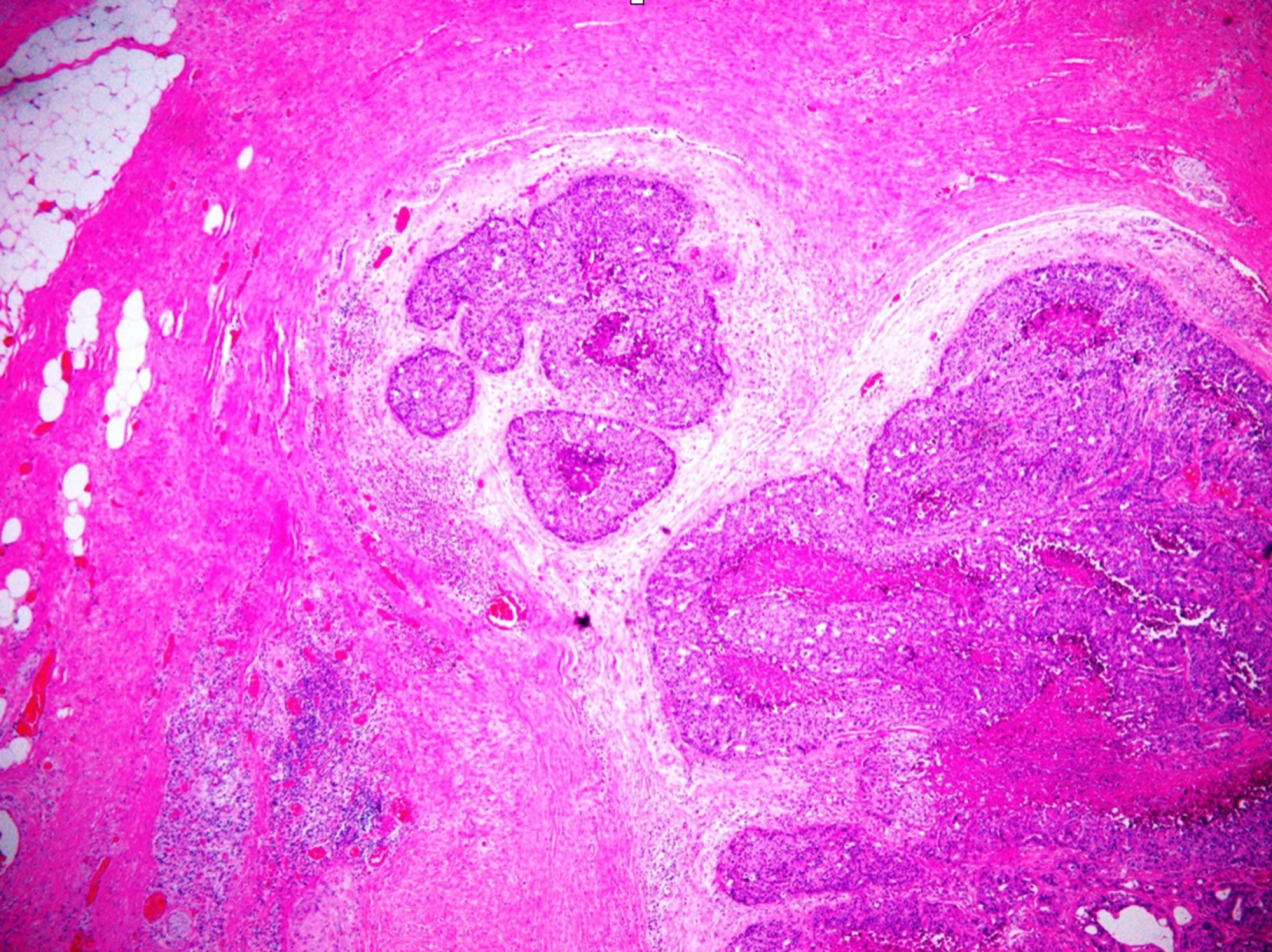
Demonstration of the formation of infiltrating glands

**Figure 7:**
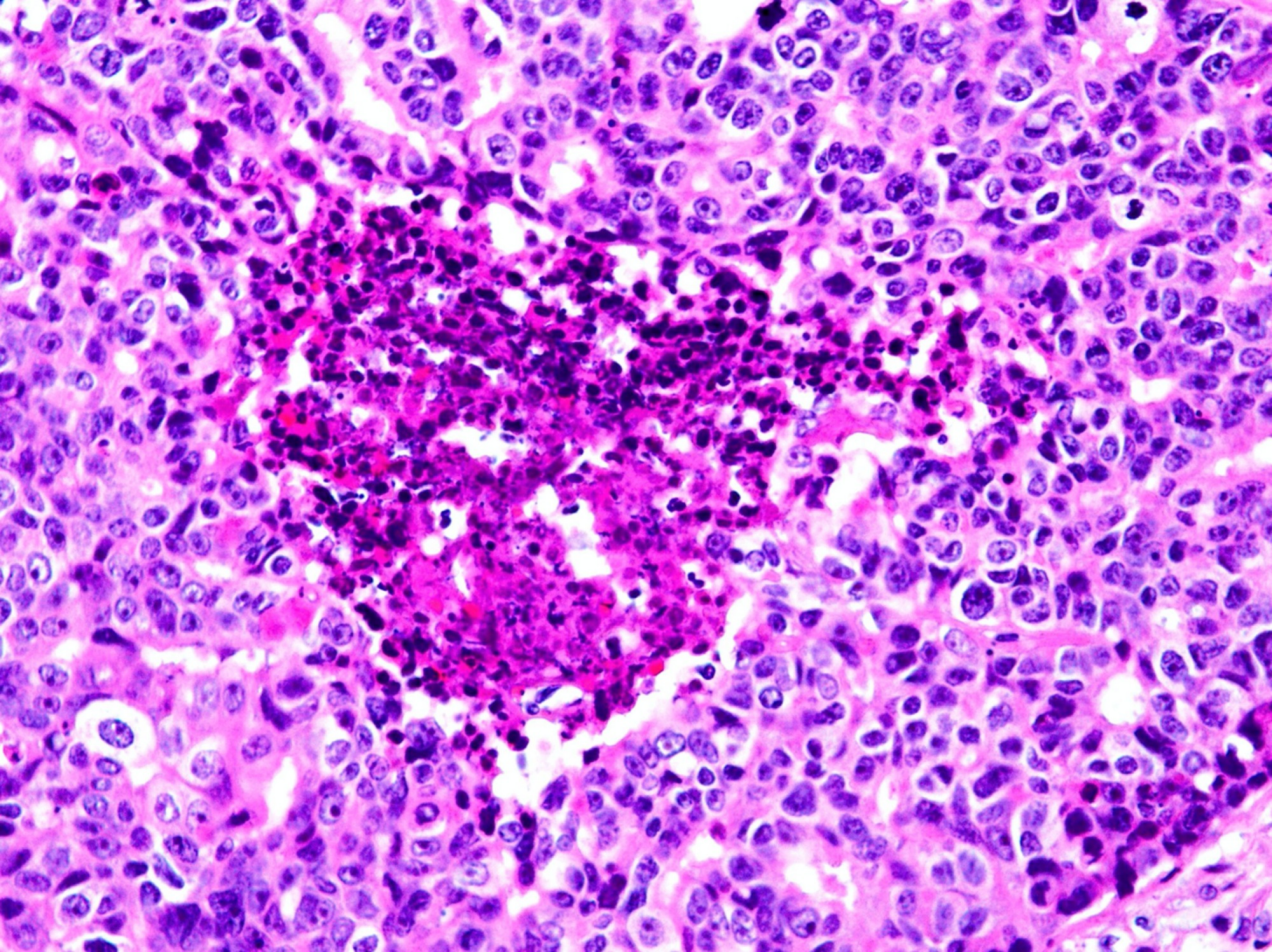
Highlighting stromal desmoplasia

In other areas, the malignant cells formed infiltrating glands with focal central necrosis, and with desmoplasia of the surrounding stroma (Figures 6-7) that comprised 30% of the estimated invasive component. Histology was consistent with ITPN with an invasive component. The duodenal wall and surgical margins were tumor-free. The distal portion of the stomach, common bile duct, gallbladder, spleen, as well as the 32 peripancreatic and six perigastric lymph nodes were all negative for malignancy. The tumor was thereby classified as Stage IIA, T3N0M0 based on the American Joint Committee on Cancer TNM staging system.

The patient had no post-operative complications. She was recommended to follow up as an outpatient with plans for adjuvant chemotherapy per guidelines for pancreatic adenocarcinoma. She was discharged to a rehabilitation facility. 

## Discussion

ITPN is a rare tumor classified by the World Health Organization (WHO) as a subtype of the premalignant intraductal tumors of the pancreas [1]. ITPN is differentiated from intraductal papillary mucinous neoplasm (IPMN), a more commonly known intraductal pancreatic tumor, due to a lack of mucin production, the presence of formation of tubules, high-grade nuclear atypia, and distinct genetic profiles [1-2]. The first case report of an intraductal neoplasm with a tubular configuration was described in 1992 in The Archives of Surgery, and was termed ‘tubular adenoma of the main pancreatic duct’ [3]. Initial reports described the tumor as intruding the pancreatic head in 84.6% of tumors (11/13 patients in the report); other findings in the original study indicated a predisposition to males (10/13) as well as a benign histologic appearance (12 out of 13 patients) [3]. Before the classification by the WHO in 2010, intraductal tumors were grouped as either IPMNs or other intraductal tubular neoplasms depending on their morphologic patterns [4]. Because ITPN has a tendency to demonstrate less aggressive behavior than other pancreatic malignancies, recognizing it as its own entity apart from other tumors, including IPMN and pancreatic ductal adenocarcinoma, is increasingly important and can be useful to clinicians.

ITPN currently comprises less than 1% of all pancreatic exocrine tumors and 3% of all pancreatic intraductal neoplasms [1]. Patients can present with nonspecific symptoms including weight loss or abdominal fullness or pain. Kim et al. reported a mean age of 56 years at diagnosis, and Basturk et al. also demonstrated a mean age at presentation of 55 years [2, 4]. Similarly to initial reports on the tumor, they are most often found in the pancreatic head at a rate of around 50%; other common sites can include the pancreatic body, or dispersed through the entire pancreas at a rate of around 15% respectively [5]. Patients tend not to be jaundiced on presentation, although some had previously experienced rounds of acute pancreatitis [2]. Upstream dilatation of the main pancreatic duct can be a finding used to specifically differentiate ITPN from IPMN [6]. ITPN tumors have a tendency to be slow-growing and can be very large at the time of diagnosis. Rooney et al. reported a range in tumor size from 1 to 15 cm, with 3 cm as the average, while Basturk et al. reported a range from 0.5 cm to 15 cm, with median size of 4.5 cm [2, 7]. The tumor size of our patient was 13.5 cm. In the cases reviewed, most patients were treated with surgery [7]. The procedures most commonly utilized were pylorus-preserving pancreatoduodenctomy and distal pancreatectomy; 18% of patients were noted to have had total pancreatectomy [1].

Histologically, ITPNs are macroscopic, tubule-forming epithelial neoplasms [7]. In comparison to IPMN, they are less cystic; one study reported less than 50% as being cystic or multicystic lesions [2]. ITPNs uniformly have high-grade dysplasia as well as frequent mitotic figures, and in comparison to the mucinous cytoplasm that comprises IPMN, mucin is found minimally in ITPN [2]. Around 40-50% of cases have the potential to be associated with invasive carcinoma, and male sex and larger tumors are associated with a high risk of invasion [1, 2]. Invasive carcinomas in relation to ITPNs consist of either individual cells or small, non-mucinous glands that extend away from the periphery of the involved ducts into the surrounding desmoplastic stroma. One study reported that in 22 of 31 patients (71%) with invasive carcinoma, the invasive component constituted less than 10% of the total tumor (55% of patients) [2].

Immunohistochemical studies can be useful to determine the ductal differentiation of pancreatic neoplasms. On review, ITPNs possess similar ductal differentiation to IPMNs and other pancreatic tumors by labeling with specific cytokeratin antibodies, such as CK7 and CK19, as well as monoclonal carcinoembyronic antigen (CEA) [8]. ITPNs can also be positive for mucin (MUC) 1 and MUC6, suggesting pancreatic (ductal) and possibly gastric pyloric gland differentiation [2, 8]. 

Postoperative survival ranges from seven months to 84 months, although data is limited on prognosis due to the rarity of the disease [1]. A literature review of 30 case reports had analyzed the prognosis in 20 of the studies and reported average survival time to be 28.6 months post-diagnosis, with most cases having no evidence of remaining disease after resection and treatment [5]. A particular case report in which a patient with ITPN and no metastases underwent pylorus-preserving duodenopancreatectomy showed survival at five months post-surgery with no evidence of remaining disease [9]. However, the prognosis of ITPN is favorable in comparison to the more commonly encountered pancreatic ductal adenocarcinomas, where median disease-specific survival was 17.0 months in a dataset of 4383 patients [10]. The patient continues to survive 14 months after diagnosis, and 11 months following pancreatectomy.

## Conclusions

ITPN is a unique tumor of the pancreas that has become increasingly recognized. It can be thought of as a tubule-forming, intraductal epithelial neoplasm that possesses high-grade dysplasia and ductal differentiation while not having significant production of mucin. Recognizing the clinical, pathologic, and immunohistochemical differences between ITPN and other intraductal and more widely recognized neoplasms such as IPMN is increasingly important even though patient presentation is often nonspecific in comparison to other pancreatic tumors. Our specific patient’s presentation was consistent with an invasive component comprising a relatively larger amount of tumor than is more commonly seen. Unlike other ductal cancers, ITPN’s association with a more favorable prognosis makes it important to recognize on a differential diagnosis. Further research is needed to understand the molecular, clinical and prognostic signature behind ITPNs, which will help provide appropriately tailored therapy and long-term follow up for these patients.
